# medplot: A Web Application for Dynamic Summary and Analysis of Longitudinal Medical Data Based on R

**DOI:** 10.1371/journal.pone.0121760

**Published:** 2015-04-02

**Authors:** Črt Ahlin, Daša Stupica, Franc Strle, Lara Lusa

**Affiliations:** 1 PhD Candidate of Statistics Programme, University of Ljubljana, Ljubljana, Slovenia; 2 Department of Infectious Diseases, University Medical Center Ljubljana, Ljubljana, Slovenia; 3 Institute for Biostatistics and Medical Informatics, University of Ljubljana, Slovenia; Huazhong University of Science and Technology, CHINA

## Abstract

In biomedical studies the patients are often evaluated numerous times and a large number of variables are recorded at each time-point. Data entry and manipulation of longitudinal data can be performed using spreadsheet programs, which usually include some data plotting and analysis capabilities and are straightforward to use, but are not designed for the analyses of complex longitudinal data. Specialized statistical software offers more flexibility and capabilities, but first time users with biomedical background often find its use difficult. We developed medplot, an interactive web application that simplifies the exploration and analysis of longitudinal data. The application can be used to summarize, visualize and analyze data by researchers that are not familiar with statistical programs and whose knowledge of statistics is limited. The summary tools produce publication-ready tables and graphs. The analysis tools include features that are seldom available in spreadsheet software, such as correction for multiple testing, repeated measurement analyses and flexible non-linear modeling of the association of the numerical variables with the outcome. medplot is freely available and open source, it has an intuitive graphical user interface (GUI), it is accessible via the Internet and can be used within a web browser, without the need for installing and maintaining programs locally on the user’s computer. This paper describes the application and gives detailed examples describing how to use the application on real data from a clinical study including patients with early Lyme borreliosis.

## Introduction

Biomedical research often involves the use of complex data that can be difficult to summarize, visualize and analyze correctly. Longitudinal data are one particular type of complex data that arises in clinical studies when the aim is to analyze the changes occurring over time; the characteristics of the patients are evaluated several times at different time points and often a large number of variables are measured at each evaluation. For example, Stupica et al [1] analysed the differences of erythema migrans (EM, early Lyme borreliosis) patients with either positive or negative *Borrelia burgdorferi* sensu lato skin culture, using a sample of 225 adult patients. Overall, more than 30 variables were recorded for each patient at each of the evaluations, which were conducted at baseline (diagnosis), 14 days, 6 and 12 months after treatment.

Data entry and manipulation of longitudinal data can be performed using spreadsheet programs like MS Excel or Open Office Calc, which include some data plotting and analysis capabilities but are not designed for the visualization and analysis of this type of data. Moreover, non-statisticians may have limited knowledge of statistics and ignore the methods that are appropriate for the analysis of longitudinal data. Existing specialized statistical software offers more flexibility and capabilities and thus has several advantages. For example, the open-source R statistical environment [2] can perform almost any state-of-the-art data analysis; it can be used to obtain figures suitable for publication and includes tools that facilitate reproducible research [3, 4]. Because of its flexibility, R is very popular among biostatisticians. Users with biomedical background, however, may find its use difficult and off-putting at first, or are even intimidated by its command line interface and lack of pull-down menus for data import and analysis [5].

Interactive web applications are a possible solution to facilitate the researchers who wish to analyze their data but do not have programming skills or experience with statistical software. To the best of our knowledge, currently a user friendly web application devoted to longitudinal data analysis is not available. Simple and interactive web-based tools that address similar needs for other types of data exist: OpenEpi [6] includes a collection of web tools for epidemiological research, the Statistics Online Computational Resource web page [7] is a repository of online statistical tools and interactive applets for simple data analysis. Web applications including complex methods of analysis were made available for genomic data: the Galaxy platform [8] provides analysis and publishing tools for scientists working in computational biology that do not have programming experience, waviCGG [9] can be used for the interactive analysis of array CGH data, iCanPlot [10] is a web tool for interactive visual data exploration of high-throughput genomic data. Common to all this applications is the attempt to bring statistical analysis closer to a broader audience of non-statisticians and to allow users to explore their data through the web browser as the user interface. A solution that is capable of running remotely is acceptable and probably preferred, since it removes administration tasks from the user and also promises greater scalability in terms of possible hardware resources (e.g., running on a dedicated high capacity remote server or in a cloud computing environment).

In this paper we present the implementation and main characteristics of medplot, the web application that we developed to facilitate the exploration and analysis of longitudinal data. The application was developed for biomedical researchers with limited experience in data analysis who wish to summarize, visualize and analyze data where numerous variables are evaluated at multiple occasions for a group of subjects. medplot is based on sound and well detailed statistical methods and can be used to prepare tables and graphs ready to be included in publications. medplot is freely available and open source, it has an intuitive graphical user interface (GUI), it is accessible via the Internet and can be used within a web browser, without the need for installing and maintaining programs locally on the user’s computer. Moreover, first time users can easily explore the features of medplot using the longitudinal data set of erythema migrans patients [1] (EM data set) included in the application.

## Design and Implementation

To reach our aim of making a flexible and user-friendly tool for complex data exploration and analysis we developed medplot, a web application based on packages and functions available in the R statistical language. medplot can be used in two different ways: (i) through the Internet, using a web application hosted on a web server (available at http://shiny.mf.uni-lj.si/medplot/) or (ii) running the web application locally. The web applications accessible through the Internet and locally display the GUI for data analysis in a web browser and are functionally identical. The R functions on which the application is based are collected in the medplot R package and can also be used within R by users familiar with the R environment (the main functions included in the package are described in the S1 Table). More details regarding the accessibility and the instructions on how to use the web application locally are given in section Availability.

The web application was developed using the framework offered by the shiny R package [11], which considerably simplifies the creation of web applications based on code written in R. The users’ data and selections are sent to a web server, which passes them to R, which in turn performs the analyses and returns the results to the web server. In practice, the user provides all the inputs and receives all the outputs in the GUI of the web browser and does not need to interact with R.

**Fig 1:**
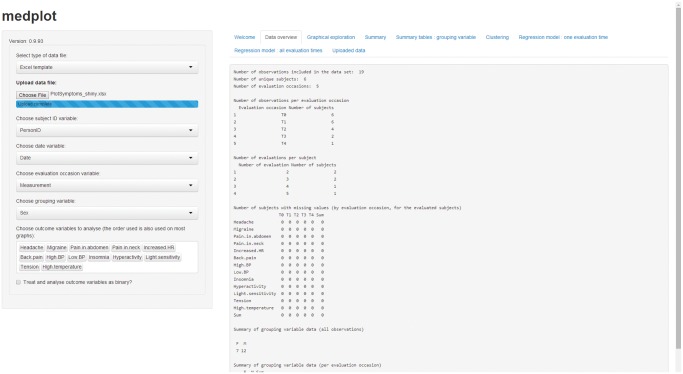
Graphical user interface in the web browser. The screen is divided in two parts: the sidebar (left part, used to for inputs) and the main panel (right part, used for outputs). The outputs are accessible through various tabs on top of the main panel part of the screen.

The GUI consists of a sidebar panel for inputs and a main panel for outputs (Fig 1). The sidebar is used to upload the data and select the variables to analyze (possible selections are described in S2 Table); the main panel consists of tabs in which the outputs are displayed. The web application supports reacting to user inputs: whenever the user changes some of the inputs via the web browser, the changes are detected, the analyses are performed again and the updated results are displayed in the browser.

medplot includes a demo data set (EM data set) that can be used to explore its features; the outputs (figures and tables) referenced in this article were obtained analyzing the demo data. We refer the reader to the Results section for detailed descriptions of the outputs.

### Data structure

Our application was designed for longitudinal data with multiple outcomes, where subjects are evaluated at multiple occasions and many variables are recorded at each occasion. Data must be organized using the so called *long format*, where each row in the data set reports the measurements (outcomes) obtained in a single occasion for a subject [12]. Three variables must appear in the data file: (i) subject ID variable (that uniquely identifies the subject being measured and assures that the measurements from the same subject are correctly identified), (ii) date variable (that indicates the date of evaluation) and (iii) evaluation occasion variable (that indicates the order of evaluation, and can be either categorical or numerical). Additionally, at least one outcome variable must also be present in the data set, to make the analysis possible. After data upload, the GUI can be used to choose the role of each variable in the uploaded data set (Fig 1).

**Fig 2:**
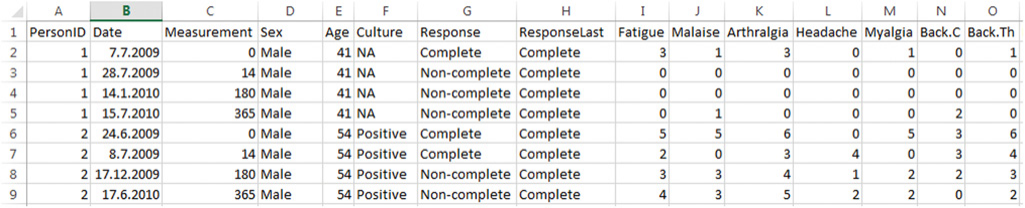
Data for first two patients of the demo data set. Data for two Erythema migrans patients are displayed. It spans eight rows, as each of them was evaluated on four occasions. Not all recorded variables are displayed.

For illustration, the rows of the EM data set referring to the first two patients are displayed in Fig 2 (limited to a subset of the variables); the complete data set is also available (https://github.com/crtahlin/medplot/blob/master/inst/extdata/DataEM.txt). The information regarding both patients (identified with PatientID 1 and 2) spans over eight rows, as each of them was evaluated four times; the Date and Measurement variables provide the exact date of evaluation and the evaluation occasion. Some of the additional variables do not change over time (like sex, age, culture), while others were measured at each evaluation occasion and are time-varying (like the intensity of the symptoms).

The variables to analyze (or outcome variables) can be numerical or binary (numerical variables can be dichotomized using medplot, specifying a threshold value). Additionally, a binary grouping variable can be specified, which is used to define two groups of subjects that are summarized separately or are compared when using some of the tools included in the application.

More details about data preparation, template files and supported formats are provided in the supplementary S1 Text. Template files examples are also visible in S16 Fig.

### Main features included in medplot

medplot can be used with numerical or binary outcomes: the methods used and the outputs provided for numerical and binary outcomes can differ. The summary and analysis tools included in the application are accessible in different tabs appearing in the main panel and can be used after the data have been uploaded. The tabs can be categorized in three groups: (i) summary tabs, (ii) graphical exploration tabs and (iii) analysis tabs, mostly reporting the results obtained using regression models.

A summary of the contents of each of the tabs is reported in Table 1. The contents of some of the tabs refer to specific evaluation occasions (selected by the user) or use the grouping variable.

**Table 1:** Main panel: tabs with statistical output.

**User interface tab**	**Description**	**Numerical outcomes**	**Binary outcomes**
**Data overview**	Basic overview and summary of the data.	Number of: observations in the data set, unique subjects, subjects evaluated and missing values at each evaluation occasion, subjects stratified by the grouping variable.	
**Graphical exploration**	Graphical exploration analysis tools.	Profile plots, Lasagna plots, Boxplots, Timeline plots	Lasagna plots, Timeline plots, Barplots
**Summary**	Graphs and tables with summary statistics of the outcome variables.	Medians (with 95% confidence intervals) and interquartile ranges	Proportions (with 95% CI) and number of subjects with positive outcomes for binary outcomes.
**Summary tables: grouping variable**	Graphs and tables with summary statistics for two groups defined by a binary grouping variable.	The two groups are compared with Mann-Whitney test	The two groups are compared with a two-sample test for equality of proportions with continuity correction.
		Unadjusted and adjusted *P* values; *Q* values for the estimation of the False Discovery Rate.	
**Clustering**	Graphical display of hierarchical clustering results for a particular evaluation occasion.	Hierarchical clustering of the outcomes, pairwise Spearman’s correlations between outcomes, visualization of the complete data (rearranged using the grouping of outcomes and subjects obtained by their hierarchical clustering).	
**Regression model: one evaluation time**	Estimates univariate regression models for a particular evaluation occasion; the covariate included in the models can be numerical or categorical.	Estimates of slope coefficients obtained with univariate linear regression with their 95% confidence intervals and P values. Numerical covariates can be modelled flexibly using restricted cubic splines.	Estimates of odds ratios obtained with univariate logistic regression with their 95% confidence intervals and P values. Firth correction can be used.
**Regression model: all evaluation times**	Estimates mixed-effects regression models, allowing a different (random) intercept for each subject. Three types of models can be estimated: with a single covariate, with a covariate and evaluation occasion, with a covariate and time since first evaluation.	Uses linear regression mixed models; provides the estimated slopes with their 95% confidence intervals and P values.	Uses logistic regression mixed models; provides the estimated odds ratios with their 95% confidence intervals and P values.
**Uploaded data**	A table with the uploaded data (or Demo data, if selected).		

The outputs consist of tables and graphs. The content of most tables can be sorted in the web browser; the tables and graphs (in Portable Network Graphics format, PNG) can be copied from the web browser and pasted into word processing programs, where they can be edited. The figures can also be saved in a vector format (Encapsulated PostScript format, EPS) using the download buttons. Most graphs are generated using functions included in ggplot2, a plotting system for R based on the grammar of graphics [13]. All the outputs are accompanied by a brief description of the method used to obtain them.

#### Summary tabs

**Fig 3:**
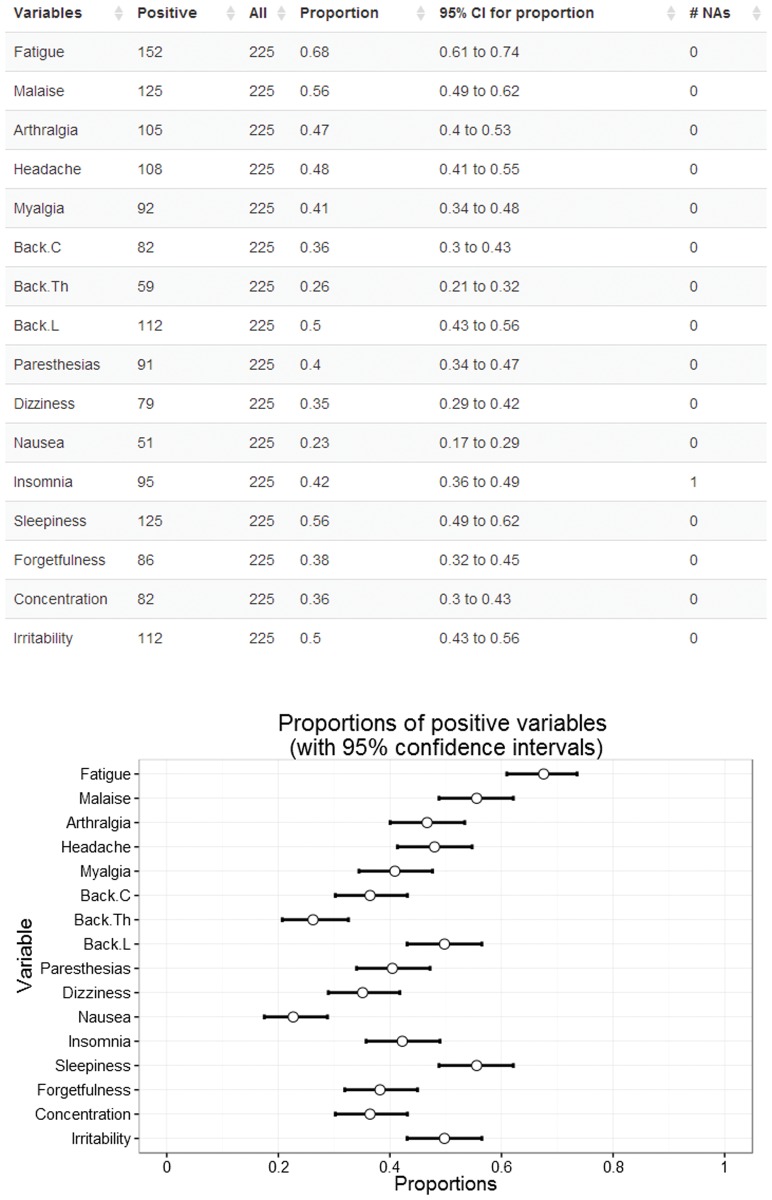
Summary tab output for binary variables. The table displays the descriptive statistics for the presence of each symptom; the plot shows the observed proportions of patients that report the presence of the symptom, along with their 95% confidence intervals.

The summary tabs report the main characteristics of the data. The basic summary statistics for the uploaded data are displayed in the *Data overview tab* (number of measurements, number of unique subjects, etc.) (S1 Fig); the main descriptive statistics for the outcome variables, summarized at each evaluation occasion, are displayed in the *Summary* tab (Fig 3, S2 Fig). Numerical variables are summarized using medians (Me) and interquartile range (IQR), while binary variables are summarized with frequencies and proportions; the number of missing values for each variable is always explicitly stated. The tables also report the 95% confidence intervals (CI) for medians (based on percentile bootstrap with 2000 iterations) or proportions (based on exact binomial method), which are also graphically displayed.

**Fig 4:**
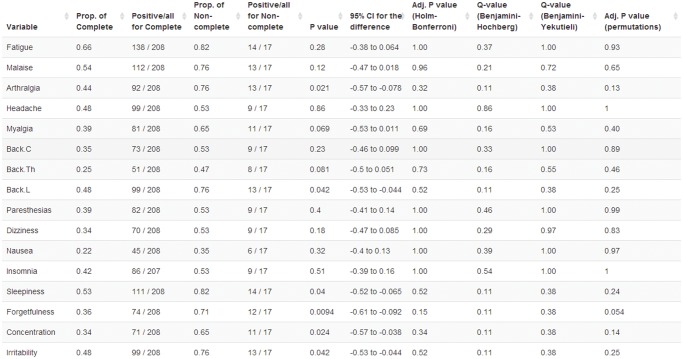
Summary: grouping variable tab output for binary variables. The table displays the summary statistics for the presence of symptoms at baseline for groups defined by the response to treatment at last evaluation. The proportions are compared, unadjusted and adjusted *P* values and *Q* values are provided (see text for details).

The groups defined by the dichotomous grouping variable are described and compared in the *Summary: grouping variable* tab (Fig 4, S3 Fig). The statistical comparisons between the two groups are performed using Mann-Whitney test for numerical variables and chi-squared test with continuity correction for binary variables.

When multiple outcomes are analyzed simultaneously with hypothesis testing, a pre-specified level of significance *α* = 0.05 does not provide appropriate control of the Type I error rate: the family wise error rate (FWER, i.e. the probability of finding at least one false positive association) approaches 1 for moderate to large number of hypotheses. For example, FWER exceeds 0.50 if more than 13 outcomes are analyzed (assuming that none of the outcomes was associated with the response and that the outcomes were independent).

medplot (Fig 4, S3 Fig) provides adjusted *P* values based on the Holm-Bonferroni adjustment (which is conservative and lacks statistical power if the outcomes are correlated), or based on a multivariate permutation based adjustment [14], a computationally intensive procedure that takes into account the correlation between outcomes and is generally more powerful than Holm-Bonferroni procedure. The adjusted *P* values are compared to the selected level of significance *α* to control probabilistically the FWER (the hypotheses are rejected if *P* < *α*, which guarantees that FWER < *α*). Moreover, medplot evaluates the false discovery rate (FDR, the expected proportion of false positive results), an alternative error control criteria [15]. The results are presented using *Q* values, the FDR analogue of the *P* values, which are the minimum FDR at which the test may be called significant. The *Q* values are evaluated using the Benjamini–Hochberg procedure (BH, [15], which assumes independent or positively dependent tests) and the Benjamini–Hochberg–Yekutieli procedure (BY, [16], which makes no assumptions about test dependencies but is more conservative than the BH procedure).

#### Graphical exploration tabs

Graphical exploration can provide many insights when data are complex, but longitudinal data are often displayed in non-optimal or misleading ways. For this reason, we devoted a lot of attention to graphical displays, by including graphs that can be used to explore various aspects of the data.

Using the *Graphical exploration* tab the user can visualize the distribution of the numerical outcomes and their changes over time with box and whisker plots (*boxplots*), profile plots (*spaghetti plots*), heat maps (*lasagna plots*) or *timeline* plots. Binary outcomes can be displayed with heat maps, bar plots and timeline plots. Each of these plots conveys different aspects of the data and its usefulness depends on the data being analyzed. The boxplots display the distributions with quartiles and the extreme values (S4 Fig); the user can decide to display in the same horizontal panel (facet) the boxplots for the same outcome (*Variables∼Evaluation occasions*) or from the same evaluation occasion (*Evaluation occasion∼Variables*).

**Fig 5:**
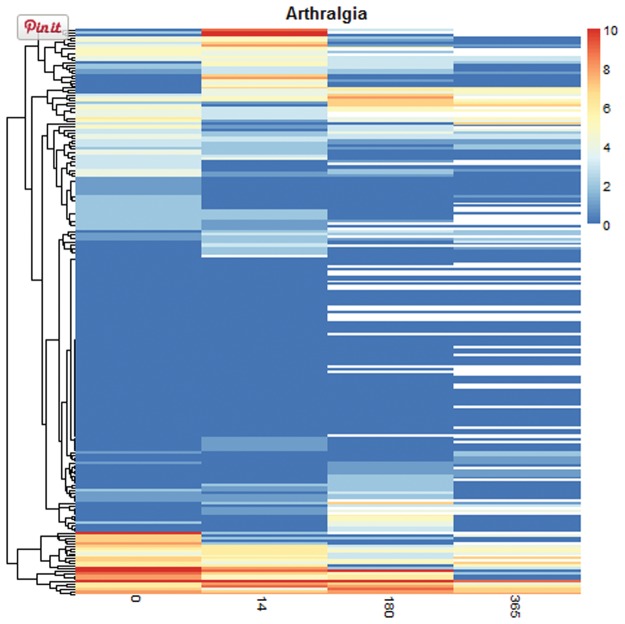
Graphical exploration tab output for numerical variables—lasagna plot. The heat map displays graphically the intensity of arthralgia for each patient (horizontal axis) and evaluation occasion (vertical axis). A dendrogram showing patient similarity is plotted on the vertical axis.

The use of profile plots and heat maps was advocated in longitudinal studies for the identification of trends and to display individual changes [12, 17], which are not visible with boxplots. Profile plots are scatterplots displaying the evaluation times and the values of the variables, where the values from the same subject are connected (S5 Fig). However, they are less useful when many subjects are plotted together and the profiles overlap, obscuring the trends. To overcome this problem medplot includes the possibility of displaying a random subset of the subjects (S6 Fig) or multiple plots for each variable. Alternatively, heat maps can be used for large data sets: evaluation times are reported horizontally, as in the profile plots, but the measurements of each subject appear in the same row and colors are used to display the value of the variables (Fig 5). In our implementation, the subjects are arranged using a hierarchical clustering algorithm (with Euclidean distance and complete linkage agglomeration method). The rearrangement of the subjects is useful for data exploration because similar subjects are grouped together.

The *timeline* plot (S7 Fig) provides a graphical display of the measurements that is similar to heat maps, where the values of the variables are displayed using dots of different sizes (*bubbles*) instead of colors. This plot is useful for displaying multiple outcomes at the same time; the interactivity of the application allows the user to select all or just a subset of the outcomes. The dates of measurement, the evaluation occasions or the number of days since inclusion in the study can be displayed on the horizontal axis.

**Fig 6:**
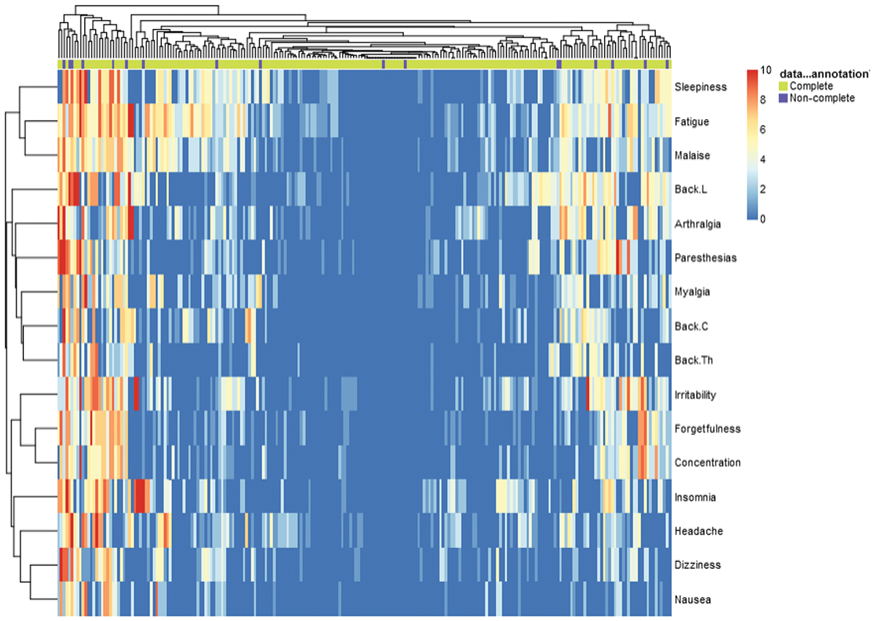
Clustering tab output—heat map displaying the similarities of reported symptoms and of patients. The colors represent the intensity of the symptoms at baseline (rows) for each patient (columns). Hierarchical clustering is used to group symptoms and patients.

The *Clustering* tab visualizes the similarity of the outcomes and of the subjects using dendrograms obtained by hierarchically clustering the outcomes (S8 Fig) and with heat maps that display the complete data obtained at a given evaluation occasion (Fig 6). The pairwise Spearman’s correlations between the outcomes are displayed graphically (S9 Fig).

#### Analysis tabs

In medplot the association of the outcomes with other variables (covariates) is estimated using regression models. The application uses linear regression models for the analysis of numerical outcomes and logistic regression models for binary outcomes; a separate model is fitted for each outcome. The analyses can be performed for a specific evaluation time (*Regression model: one evaluation time* tab) or using all the data (*Regression model: all evaluation times* tab); in the latter case the repeated measurements for each subject are taken into account using mixed-effects regression models, where subjects’ effects are treated as random variations around a population mean (including random intercepts in the model) [18].

The mixed-effects models can include a single covariate chosen by the user (choosing *Outcome ∼ Covariate + Subject (random effect)*), a covariate and the evaluation occasion (*Outcome ∼ Covariate + Evaluation occasion + Subject (random effect)*), or a covariate and time from inclusion (*Outcome ∼ Covariate + Time from inclusion + Subject (random effect)*). All the covariates are modelled as fixed effects. The second and third model adjust the analysis for the evaluation time but they differ in the way in which time is modelled: the evaluation occasion is modelled as a categorical covariate (using baseline evaluation as the reference category), while time from inclusion (defined as the number of days from first evaluation) is modelled as a numerical covariate. The most appropriate choice depends on the data being analyzed: time from inclusion should be preferred when it is sensible to assume a linear relationship between time and the outcomes (on the logit scale for the logistic models).

The results are reported as estimated regression coefficients—slope coefficients (beta) for linear regression or odds ratios (OR) for logistic regression—with their 95% CI are displayed using tables and graphs. The association between the outcomes and evaluation occasion, without adjusting the analysis for other covariates, can be evaluated using the *Outcome ∼ Covariate + Subject (random effect)* model, selecting the evaluation occasion as a covariate.

**Fig 7:**
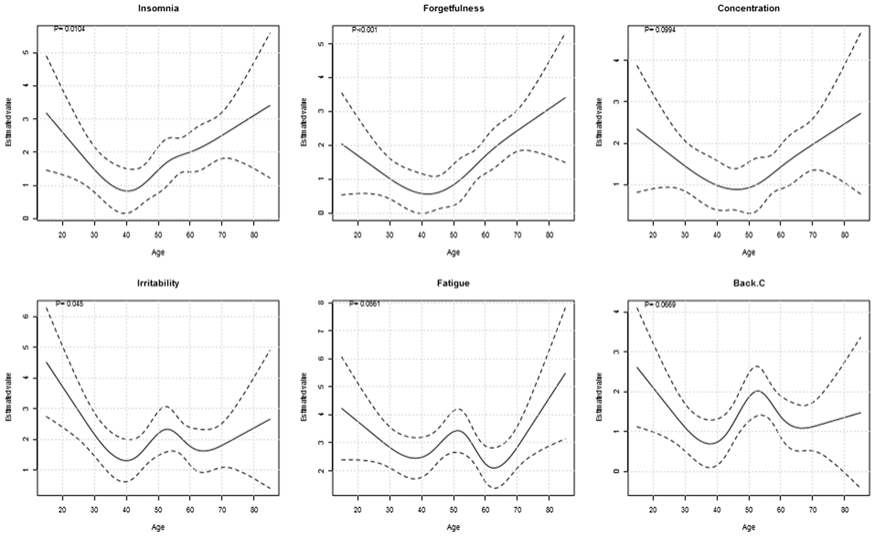
Regression model: one evaluation time tab output—estimation of non-linear associations. The graphs display the estimated associations between the age of the patients and selected symptom intensities at baseline evaluation. Restricted cubic splines are used for modeling. See text for details.

For the analysis of data at a specific evaluation time it is possible to flexibly model the association of numerical covariates with the outcomes (using restricted cubic splines [19]) and to display graphically the estimated shape of the relationship between the continuous covariates and the outcomes (with 95% CI) (Fig 7). medplot also includes the Firth’s correction for logistic regression models [20], which is useful for small data sets [21] or when the phenomenon of separation occurs (the responses and non-responses can be perfectly separated by a covariate and as a consequence the parameter can not be estimated and the estimate diverges to infinity). These situations occur often in small samples or when the covariates or outcomes are highly imbalanced.

Users interested in comparing the means of numerical outcomes between two groups of patients at an evaluation time can use the *Regression model: one evaluation time* tab instead of the *Summary: by grouping variable* tab (where the distributions are compared with Mann-Whitney tests). In this setting the slope coefficients can be interpreted as estimated mean differences and the *P* values are identical to those obtained with a t-test with equal variances.

## Results

In this section we illustrate the use of medplot through the analysis of the demo data, which are briefly described.

### Demo data: the erythema migrans (EM) data set

The demo data set is a real data set from a study on erythema migrans (early Lyme borreliosis) where the clinical, epidemiological and microbiological characteristics of 225 adult patients were evaluated at baseline, 14 days, 2, 6, and 12 months after treatment [1]. Patients were asked to complete a written questionnaire, asking whether they had had within the preceding week any of 16 nonspecific symptoms: fatigue, malaise, arthralgias, headache, myalgias, paresthesias, dizziness, nausea, insomnia, sleepiness, forgetfulness, concentration difficulties, irritability, or pain in the lower, thoracic or cervical spine. The severity of any individual symptom was graded by the patient on a 10-cm visual analog scale (0: absent, 10: most severe). The other variables included in the analysis were: patient’s gender, age (in years), positivity of *Borrelia burgdorferi* sensu lato skin culture, complete response to treatment at each evaluation and complete response to treatment at last available visit. Complete response was defined as resolution of erythema migrans with return to pre-Lyme borreliosis health status. In the examples included in the paper we focus on the variables describing the presence and intensity of nonspecific symptoms as reported by the patients, and use them as outcome variables.

The demo data can be used in the application selecting *Demo data* in the *Select type of data file* drop down menu in the sidebar. The data is displayed in the *Uploaded data* tab.

#### Summary tabs

The *Data overview* tab reports that the EM data set contained 812 observations from 225 unique subjects, obtained in 4 evaluation occasions (S1 Fig); 166 subjects were evaluated 4 times, 29 subjects three times and 30 two times; 100 males were included in the sample. Among the evaluated subjects the outcomes were missing for only two records. The *Summary* tab, which summarizes the outcomes, reports that at baseline most symptoms had a median intensity equal to 0; fatigue had the highest median intensity and variability (Me = 2, interquartile range (IQR): 0 to 5, 95% CI for the median: 2 to 3)(S3 Fig). Using the uploaded data, we could define the binary outcomes that describe the presence or absence of the symptoms (selecting *Treat variables as binary* and choosing a threshold equal to 0 in the sidebar panel); after the selection all the outputs displayed in the main panel are based on the new outcomes. For example, the updated *Summary* tab reports the number and proportion of patients presenting the symptoms rather than the medians and IQR (Fig 3). The new table shows that fatigue was the most commonly reported symptom (reported by 152/225 (68%) patients, 95% CI: 61% to 74%), followed by sleepiness, malaise and lower back pain; nausea was the least common symptom.

The description and comparison of the outcomes at baseline, categorized by the response to treatment at last available visit is available in the *Summary: grouping variable* tab (selecting: ResponseLast as grouping variable in the sidebar, and Evaluation occasion = 0 in the tab, S3 Fig). From this analysis it emerged that the patients with non-complete response at last available visit (n = 17) reported higher intensity of the symptoms at baseline compared to those that achieved a complete response (*P* < 0.05 for 9 out of the 16 symptoms). The multiplicity problem, caused by the 16 statistical tests that were performed, was controlled selecting the *Calculate P value adjustment* option (which adds the last four columns to the table in S3 Fig).

With the Holm-Bonferroni adjustment only the intensity of arthralgia, lower back pain, sleepiness and forgetfulness remained significantly associated with complete response (adjusted *P* < 0.05, S3 Fig); a similar result was obtained interpreting the adjusted *P* values obtained with the multiple permutation procedure. Using the FDR approach (based on BH procedure) arthralgia, myalgia, sleepiness, forgetfulness, concentration disturbances, thoracic and lower back pain had *Q* < 0.05: in this list of symptoms we would expect less than 5% of false positives, i.e. less than one (7 ⋅ 0.05 = 0.35) false positives result. Alternatively, setting the threshold to *Q* = 0.07 we would obtain a list of 11 symptoms where we would expect less than 1 false positive result. The *Q* values obtained using the BH procedure should be preferred to the BY *Q* values in this application because it is reasonable to expect a positive dependence among the test results. Analyzing the presence rather than the intensity of symptoms the differences between the groups were more difficult to demonstrate (Fig 4). The unadjusted analysis identified 6 symptoms (*P* < 0.05), the Holm-Bonferroni approach did not identify any (minimum adjusted *P* = 0.15), the multivariate permutation approach identified with 95% confidence only the presence of forgetfulness; with the FDR-BH approach the smallest *Q* value was 0.11: using this threshold we would identify 5 symptoms and expect less than 1 false positive result among them (0.11 ⋅ 5). The discrepancy between the analyses based on the intensity and the presence of symptoms is not surprising: the statistical comparisons based on dichotomized numerical variables usually have smaller statistical power than those based on the original data [22].

#### Graphical exploration tabs

Different aspects of the EM data set could be recognized using the *Graphical exploration* tab. The boxplots made clearly visible that the intensity of the symptoms tended to decrease at successive evaluations (S4 Fig): an exception was lower back pain, which was frequent at baseline but did not decrease as markedly as the other symptoms over time. The individual changes were conveyed more effectively using the lasagna plots (Fig 5) rather than the profile plots (S5 Fig and S6 Fig); this was expected because of the relatively large sample size of the EM data set and because of the small number of possible values for the intensity of the symptoms (0-10). Lasagna plots were useful for visualizing missing data (displayed with white color in our plot); in the EM data set most missing values appeared at third and fourth follow-up visits. Specific time patterns and subgroups of patients with similar characteristics could be identified with the lasagna plots. For example, most subjects did not report arthralgia during the follow-up. The subset of subjects that reported high intensity of arthralgia at baseline tended to report it also at successive evaluations; another subset of subjects presented an increase of the symptom after 2 weeks of treatment but in most cases the symptom was not reported at later follow-up visits.

The timeline plot (selecting *Time from inclusion* on the horizontal axis) was useful for visualizing the adherence to the protocol in the EM data set: as expected, not all the follow-up visits were performed exactly at the scheduled times (14, 180 and 365 days after enrollment) but the differences between the planned and effective dates were small in most cases (S7 Fig). In the *Clustering* tab we observed that the intensities of symptoms were positively correlated (S9 Fig); the pairs of symptoms with the largest Spearman’s correlation were forgetfulness and concentration disturbances (rho = 0.73), and fatigue and malaise (rho = 0.72), while sleepiness and insomnia had the smallest correlation (rho = 0.16). In the hierarchical clustering dizziness, nausea, headache and insomnia were grouped together at baseline (S8 Fig). Within the other group of symptoms other three subgroups could be identified: (i) arthralgia and pain in the lower, thoracic or cervical spine, (ii) fatigue, malaise and myalgia and (iii) sleepiness, concentration disturbances and paresthesias. Using the heat map that displays all the data at baseline (Fig 6) a subgroup of patients that reported the presence of most of the symptoms could be identified (left part of the figure). However, the dendrogram and heat maps obtained by clustering should not be over-interpreted, as clustering analysis is only exploratory and can be very sensitive to small changes in data. Results can also markedly differ when using different clustering algorithms (with different distances or agglomeration methods), producing different grouping of the variables or of the subjects.

#### Analysis tabs

The mean intensity of the symptoms at baseline between responders and non-responders was compared using the *Regression model: one evaluation time* tab (selecting *ResponseLast* as covariate). A separate regression model was estimated for each of the symptoms. On average, the non-responders reported larger mean intensities of the symptoms at baseline compared to responders: the mean differences were between 0.9 (for nausea, 95% CI for the difference: 0.05 to 1.8) and 2.6 (for arthralgia, 95% CI: 1.4 to 3.8) (S10 Fig).

On average, older patients reported higher intensity of arthralgia, lower back pain and forgetfulness, and lower intensity of sleepiness and nausea (S11 Fig, results obtained selecting *Age* as covariate). The expected difference in the intensity of lower back pain between two patients with 10 years of difference in age was 0.4 (beta = 0.04, the regression coefficients can be interpreted as the estimated mean difference in intensity for two patients that differ by one year of age).

We assessed whether the increase of average intensity of the symptoms with age was linear by fitting linear regression models that did not assume the linearity between age and the outcomes (selecting *Use flexible model of the association of the selected variables with the numerical covariate* in the tab) (S12 Fig). The linearity assumption did not seem appropriate for many of the symptoms (Fig 7, S12 Fig). For example, a U-shaped association was estimated for forgetfulness, concentration disturbances, irritability and insomnia (higher average intensity for youngest and oldest patients). The youngest patients had the highest intensity of nausea, which decreased with age until the age of 40 and remained rather stable afterwards; many symptoms exhibited a peak in their intensity around 50 years (an increase between 40 and 50 years of age, followed by a decrease).

The complete information provided by the longitudinal data was taken into account estimating mixed-effects regression models (available in the *Regression model: all evaluation times* tab). For example, the association between the evaluation occasion and the intensity of the symptoms was assessed selecting the evaluation occasion (*Measurement*) as the covariate in the *Outcome ∼ Covariate+Subject (random effect)* model (S13 Fig). Similarly as described above, a different model was estimated for each of the symptoms (which were used as the outcome variables).

For each model (symptom), three parameters were estimated for evaluation occasion (denoted with Level: 14, 180 and 365): each estimated parameter expresses the estimated average difference in the intensity of the symptom between the specified evaluation occasion and baseline, which is used as reference level. The intensity of all the symptoms tended to decrease with time (most of the estimated slope coefficients were negative), with the exception of nausea that, compared to baseline, had a higher intensity after 14 days of treatment (estimated slope for 14 days = 0.35; 95% CI: 0.11 to 0.59). Similar results were observed modelling the presence rather than the intensity of the symptoms (using a logistic regression model and dichotomizing the outcomes). For example, the odds for the presence of nausea after 14 days of treatment were 1.77 times larger compared to baseline (OR = 1.77, 95% CI: 1.02 to 3.07) (S14 Fig).

The association of the other covariates with the outcomes could be assessed by fitting the *Outcome ∼ Covariate+Evaluation occasion + Subject (random effect)* models, which adjust the analysis for the evaluation occasion. The outputs obtained for the analysis of sex showed that on average women reported higher intensity for all the symptoms. The biggest differences between sexes were observed for malaise, insomnia, fatigue, forgetfulness, arthralgia, headache and nausea (S15 Fig).

## Discussion and Future Directions

In this paper we presented medplot, a web application for the analysis of longitudinal data. medplot has an intuitive interface, it can be used via the Internet without the need to install any program locally, is freely available, open source and it includes plotting tools and analysis methods that are not commonly available to non-statisticians. medplot was developed using the shiny framework and it is based on functions available in the R statistical language. To the best of our knowledge, a tool with similar characteristics did not exist.

Longitudinal data are common in clinical research and require specialized statistical methods and software, which are both difficult to use by non-statisticians. We exemplified how to use medplot in clinical research by showing how to analyze a complex longitudinal data set of patients with erythema migrans, and provided the interpretation of the obtained results. The statistical methods included in medplot are not comprehensive and do not cover all the possible analyses of longitudinal data. However, medplot includes the most commonly needed visualization and analysis methods, including some methods that are undeservingly rarely used in clinical research.

The choice of the methods to include in medplot was influenced by our previous experience with longitudinal data analysis, where we often used statistical approaches that differed from those commonly employed in clinical papers addressing similar problems. For example, we used mixed-effects logistic regression models to assess the association between complete response and culture positivity in EM patients, adjusting the analysis for the evaluation occasion [23]. We used a multivariate permutation procedure to control for false positives associations between the presence of symptoms and pleocytosis in a cohort of suspected early Lyme neuroborreliosis patients with EM [24]. We modelled non-linearly the association between between various numerical variables and the probability of a positive culture in EM patients [25]. The aim of this project was to make available these and other methods to a broader group of non-statisticians.

Since our goal was to make the analysis tools available freely and be extendable in the future, R programming language was our obvious choice. R is free and open source, supported in all major operating systems and has a myriad of statistical functions available through its add-on packages [26]. While many other free software packages exist, including some that could be used via the browser [6–8, 27], we could find none with all the mentioned characteristics, which could also be used for the analysis that medplot supports.

The medplot package and its web applications will continue to evolve as needed with upgrades to existing plots, summaries and analysis tools. Due to the open source nature, contributions from other developers are encouraged.

## Availability

Web applications that, like medplot, use the shiny framework, should work in web browsers supporting WebSockets [28], which are all newer versions of major browsers (e.g. at least Internet Explorer 10, Mozilla Firefox 6, Google Chrome 14, Safari 6, etc.). The firewall on the user’s computer has to allow communication via WebSockets. Web applications that run on a remote server might work without this requirement.

### medplot on a remote server

The medplot application is available online on our remote server for demonstration purposes at the address http://shiny.mf.uni-lj.si/medplot/. To access and use the web application the user needs only a working Internet connection and a supported web browser.

R statistical environment runs on the remote server and the user does not need to set it up. Setting up a remote shiny server is outside the scope of this text and is described in detail in [29].

### medplot locally

The web application medplot can be used locally, but some additional requirements apply. Firstly, the user has to have the R statistical environment installed, for which instructions can be found in [30]. Secondly, the devtools R package has to be installed by running the following command in the the R console window:

install.packages(“devtools”)

Detailed instructions for installing devtools and its dependencies can be found in [31]. The installation of devtools requires the presence of some development tools on the user’s system; the tools are platform dependent and are described in the installation instructions of devtools. For example, on MS Windows Rtools has to be installed (detailed instructions can be found in [32]). Thirdly, if the user wishes to upload files created using the MS Excel template, perl has to be installed. General instructions for installation and links to different distributions of pearl can be found in [33]. For MS Windows platform, we tested the application using Strawberry perl distribution, but other distributions may also be appropriate. The users might have to reboot their computer for the installations to come into effect. We suggest that the users download and install the most recent and stable versions of R and of the other needed tools.

Then, the following commands should be entered in the R console window:

library(devtools)
install_github(“crtahlin/medplot”)

These will load the devtools package and install the medplot package. Finally, to run the application, the following commands should be entered in the R console window:

library(medplot)
medplotOnline()

A web browser should open with the web application already loaded. Only these last two commands are needed at subsequent application runs.

The medplot R package containing R code and template MS Excel files for entering data is freely available. The latest stable and development versions of the code can be downloaded from GitHub (https://github.com/crtahlin/medplot). Users can follow the “Issues” link on the GitHub site to report bugs or suggest enhancements.

## Supporting Information

S1 Text Data preparation. (PDF)

S1 Table Main medplot functions, their outputs, locations on the graphical interface and the main packages they use and depend on. (PDF)

S2 Table Sidebar: data upload and variable selection. (PDF)

S1 Fig Data summary tab output. Output on the Data Summary tab, showing basic summary of the data.
(TIF)

S2 Fig Summary tab output for numerical variables. The table displays the descriptive statistics for the intensities of symptoms at baseline (medians, interquartile ranges, 25% and 75% percentile, number of missing values), and 95% confidence intervals for the medians based on bootstrap. The medians and 95% CI are also graphically displayed.
(TIF)

S3 Fig Summary: grouping variable tab output for numerical variables. The table displays the summary statistics for the intensity of symptoms at baseline for groups defined by the response to treatment at last evaluation. The proportions are compared, unadjusted and adjusted P values and Q values are provided (see text for details).
(TIF)

S4 Fig Graphical exploration tab output—boxplots. Boxplots displaying the intensities of symptoms reported at each evaluation occasion.
(TIF)

S5 Fig Graphical exploration tab output—profile (spaghetti) plot for all subjects. The intensity of arthralgia is shown for each patient and evaluation occasion (horizontal axis). Each line connects the values for the same patient.
(TIF)

S6 Fig Graphical exploration tab output—profile (spaghetti) plot for a subset of subjects. The intensity of arthralgia is shown for a subset of 10 patients.
(TIF)

S7 Fig Graphical exploration tab output—timeline plot. Intensities of reported symptoms are represented by different sizes of circles. Each line represents a patient. The horizontal axis represents days since inclusion in the study. The size of the bubbles is proportional to the intensity of the symptoms.
(TIF)

S8 Fig Clustering tab output—dendrogram displaying the hierarchical clustering of the variables. The intensities of the symptoms at baseline evaluation are grouped using hierarchical clustering. The results are displayed using a dendrogram.
(TIF)

S9 Fig Clustering tab output—Spearman’s correlation of the variables. Graphical display of the Spearman’s correlation of the intensity of the symptoms at baseline evaluation.
(TIF)

S10 Fig Regression model: one evaluation time tab output—using linear regression and the ResponseLast variable as covariate. Estimates (with 95% CI and P values) obtained from the linear regression models where patient response at last available visit is used as a covariate and the intensity of the symptoms at baseline evaluation is the outcome variable. A separate model is fitted for each symptom; the estimated intercept of each model is also reported. The slopes and their 95% CI are also displayed graphically.
(TIF)

S11 Fig Regression model: one evaluation time tab output—using linear regression and the Age variable as covariate. Estimates (with 95% CI and P values) obtained from the linear regression models where patient age is used as a covariate and the intensity of the symptoms at baseline evaluation is the outcome variable. A separate model is fitted for each symptom; the estimated intercept of each model is also reported. The slopes and their 95% CI are also displayed graphically.
(TIF)

S12 Fig Regression model: one evaluation time tab output—non-linear modeling using restricted cubic splines with the Age variable as covariate. The graphs display the estimated associations between age (horizontal axes) and the intensities of symptoms at baseline evaluation occasion. Restricted cubic splines are used for modeling.
(TIF)

S13 Fig Regression model: all evaluation times tab output—using linear regression with Measurement variable as covariate. The table reports the estimated slopes obtained from linear regression models in which the association of the evaluation occasion (covariate) and intensity of symptoms (outcome) is evaluated. The multiple measurements from each patient are taken into account using a random intercept mixed model.
(TIF)

S14 Fig Regression model: all evaluation times tab output—using logistic regression with Measurement variable as covariate. The table reports the estimated odds ratios obtained from logistic regression models in which the association of the evaluation occasion (covariate) and presence of symptoms (outcome) is evaluated. The multiple measurements from each patient are taken into account using a random intercept mixed model.
(TIF)

S15 Fig Regression model: all evaluation times—using linear regression with Sex and Measurement variables as covariates. The table reports the estimated slopes for the effect of covariate (sex) on the outcome (intensities of symptoms). A second covariate (evaluation occasion) is included in the model. The multiple measurements from each patient are taken into account using a random intercept mixed model.
(TIF)

S16 Fig Sample data in different formats. MS Excel sheets containing demo data in different formats. The panels are: A) Excel template file showing the DATA sheet, B) Excel template file showing the PATIENTS sheet, C) tab separated values file open in Excel.
(TIF)
