# Supplementary material for: medplot: A Web Application for Dynamic Summary and Analysis of Longitudinal Medical Data Based on R
Source: PLoS One. 2015 Apr 2;10(4):e0121760. doi: 10.1371/journal.pone.0121760 (PMC4383594; doi:10.1371/journal.pone.0121760)
Supplement: S1 Text — (PDF) [file pone.0121760.s001.pdf]

Data preparation

In our application we deal mostly with longitudinal data, where subjects are measured multiple times. Three variables are mandatory and must appear (as columns) in the data file. The first is the **subject ID variable**, which uniquely identifies the subject being measured and assures that the measurements from the same subject are correctly identified. The two additional variables indicate the time when the single evaluation of the subject was conducted: (i) the **date variable** indicates the date of evaluation and (ii) the **evaluation occasion variable** indicates the order of the evaluation, and can be either categorical or numerical. Additionally, at least one **outcome variable** must also be present in the data set, to make the analysis possible. **Additional variables** are allowed in the data file. After data upload, the GUI can be used to choose the role of each variable in the uploaded data set.

Besides using the provided demo data, the users can upload their own data, which can be supplied in a: (i) tab delimited text file or (ii) MS Excel spreadsheet prepared using the templates that we make available. For reference, the tab delimited text file containing demo data is accessible at:
`https://github.com/crtahlin/medplot/blob/master/inst/extdata/DataEM.txt`

The MS Excel template file containing some example data is accessible at (click the "View Raw" link to download):
`https://github.com/crtahlin/medplot/blob/master/inst/extdata/PlotSymptoms_shiny.xlsx`

In **S16 Fig.** we show a few records of the demo data, prepared both in the tab delimited format and the MS Excel template provided with the application. See also sections Demo data and Availability in the main article for a description of the demo data and general package availability information.
For data organization we decided to use the so called *long format*, where each row in the data set reports the measurement obtained for a subject at a single evaluation occasion. The long format makes data entry of the variables measured at each evaluation occasion straightforward: each variable is reported in a single column and each row represents a single evaluation occasion. For example, there is only one column for the intensity of headache in our demo data; the information about the date of evaluation and subject ID is retrievable from other columns. Data entry for variables that are measured at each evaluation is therefore facilitated using the long format. In our demo data we have 16 such variables - the severity of different symptoms.

By design, the values of some variables (covariates) cannot change during the follow-up period. For example, in our demo data the variables of this type are sex, age at enrollment, culture positivity evaluated at enrollment, response to treatment after 6 months. The users that are preparing the data using the MS Excel templates provided with the package can specify the value of the covariates that change over time in the worksheet called **DATA**, while all the other information is entered in the **PATIENTS** worksheet. The data from both tabs is merged at import into `medplot`. In the case of a tab separated values text file, each line should contain all the variables - both those depending on evaluation occasion and those not. Note that dates should be in the "dd.mm.yyyy" format (eg. 27.12.2013), regardless of the data format used. Data that were already collected in electronic format can perhaps more easily be saved as tab delimited (TSV) text files (than entered into MS Excel), as export to TSV file functionality is widely available in spreadsheet and database programs.
