# Supplementary material for: medplot: A Web Application for Dynamic Summary and Analysis of Longitudinal Medical Data Based on R
Source: PLoS One. 2015 Apr 2;10(4):e0121760. doi: 10.1371/journal.pone.0121760 (PMC4383594; doi:10.1371/journal.pone.0121760)
Supplement: S1 Table — (PDF) [file pone.0121760.s002.pdf]

medplot R function	Output, where the functions is used, packages it uses
summarizeData()	Different basic summaries of the data used in the analysis. (on tab: `Data summary`; uses `base::summary()`)
plotDistribution()	Barplot of proportion of subjects with positive variable values. (on tab: `Graphical exploration`; uses `graphics::barplot()`)
plotTimeline()	Plot of variable intensity via circle size. Subject are on the vertical axis. The horizontal axis depends on user selection and can show: dates, time since inclusion in the study, evaluation occasion. (on tab: `Graphical exploration`; uses `ggplot2::ggplot()`)
plotTimelineBoxplots()	Boxplots showing distribution of variables on different evaluation occasions. Faceting can be done either by variables or by evaluation occasions. (on tab: `Graphical exploration`; uses `ggplot2::ggplot()`)
plotTimelineProfiles()	Profile (spaghetti) plots showing variable values over measurement occasions, for each variable in a separate facet. Three different types of graphs are possible: for all subject, a random subset of subjects, multiple graphs with a limited number of subjects each (applicable when a large number of subjects is analyzed). (on tab: `Graphical exploration`; uses `ggplot2::ggplot()`)
plotLasagna()	Lasagna plots (heat maps) of the data. (on tab: `Graphical exploration`; uses `pheatmap::pheatmap()`)
plotPropPositive()	Vertical barplot showing proportion of subjects in each group having positive outcome variables. Bars are plotted for each evaluation occasions. (on tab: `Summary`; uses `graphics::barplot()`)
tableMedians()	Returns data frame containing medians and related statistics. (on tab: `Summary`; uses `boot:boot.ci()`)
plotValueswithCIs()	Function that plots values of statistics and their confidence intervals (e.g. medians with 95% confidence intervals). (used on various tabs; uses `ggplot2::ggplot()`)
tableProportions()	Returns data frame containing proportions and related statistics. (on tab: `Summary`; uses `stats::binom.test()`)
plotPropPositiveCI()	Plot of proportions of subjects in each group having positive outcome variables - with confidence intervals. (on tab: `Summary tables : grouping variable`; uses `ggplot2::ggplot(), stats::prop.test()`)
tablePropPosGroups()	Table containing proportion of subjects with positive variable values and other related statistics for both groups. (on tab: `Summary tables : grouping variable`; uses `stats::prop.test(), stats::wilcox.test(), stats::p.adjust()`)
tableMeGroups()	Table containing the medians of values and other related statistics for both groups. (on tab: `Summary tables : grouping variable`; uses `stats::wilcox.test(), stats::p.adjust()`)
plotDendrogram()	Dendrogram for the similarity of the variables. (on tab: `Clustering`; uses `stats::hclust()`)
plotClusterHeatmap()	Heat map displaying subjects on the horizontal axis and selected outcome variables on the vertical axis. Dendrograms are ploted at both axis. User can select additional variables for annotating the graph. (on tab: `Clustering`; uses `pheatmap::pheatmap()`)
plotCorrelations()	Heat map displaying correlations of selected outcome variables. (on tab: `Clustering`; uses `pheatmap::pheatmap()`)
tableLogistf()	Table containing results of logistic regression with Firth correction. (on tab: `Regression model : one evaluation time`; uses `logistf::logistf()`)
tableLogist()	Table containing results of logistic regression. (on tab: `Regression model : one evaluation time`; uses `stats::glm()`)
tableLinear()	Table containing results of linear modeling. (on tab: `Regression model : one evaluation time`; uses `stats::glm()`)
plotRCS()	Plot of flexible modeling of the variables via restricted cubic splines (RCS). (on tab: `Regression model : one evaluation time`; uses `stats::glm(), rms.trans::rcs(), stats::anova()`)
tableRCS()	Table of results of modeling with the restricted cubic splines. (on tab: `Regression model : one evaluation time`; uses `stats::glm(), rms.trans::rcs(), stats::anova()`)
mixedModel()	Function that returns a list of results of mixed-effects modeling. (on tab: `Regression model : all evaluation times`; uses `lme4::glmer(), lmerTest::lmer()`)
