# Supplementary material for: medplot: A Web Application for Dynamic Summary and Analysis of Longitudinal Medical Data Based on R
Source: PLoS One. 2015 Apr 2;10(4):e0121760. doi: 10.1371/journal.pone.0121760 (PMC4383594; doi:10.1371/journal.pone.0121760)
Supplement: S2 Table — (PDF) [file pone.0121760.s003.pdf]

Selection	Description
Select type of data file	Format of data to upload. Possible choices are tab separated values text file (TSV) or MS Excel (the provided template must be used). If the user selects the option Demo data, the erythema migrans data set provided with the `medplot` package is loaded and default parameter selections are made.
Upload data file	File containing the data to upload.
Choose subject ID variable	Name of the variable in the data that uniquely identifies the subjects.
Choose date variable	Name of the variable in the data that contains the dates of measurements. The dates must be provided using the `dd.mm.yyyy` format.
Choose evaluation occasion variable	Name of the variable in the data that identifies the evaluation occasion. It is treated as a categorical variable.
Choose grouping variable	Name of the binary variable in the data that defines two subgroups of subjects that the user is interested in comparing.
Choose outcome variables to analyse	Names of the variables that will be analyzed. Multiple variables can be selected.
Treat and analyse outcome variables a binary (as in presence or absence of a symptom).	Selection that defines if the outcome variables should be dichotomized and considered binary.
Threshold for positivity of the outcome variables	Threshold value used to dichotomize the outcome variables. The groups are defined as below or equal to the threshold, or above the threshold. This option is visible only if the user selects to analyse the outcome variables as binary.
