# Supplementary figures and images for: medplot: A Web Application for Dynamic Summary and Analysis of Longitudinal Medical Data Based on R
Source: PLoS One. 2015 Apr 2;10(4):e0121760. doi: 10.1371/journal.pone.0121760 (PMC4383594; doi:10.1371/journal.pone.0121760)

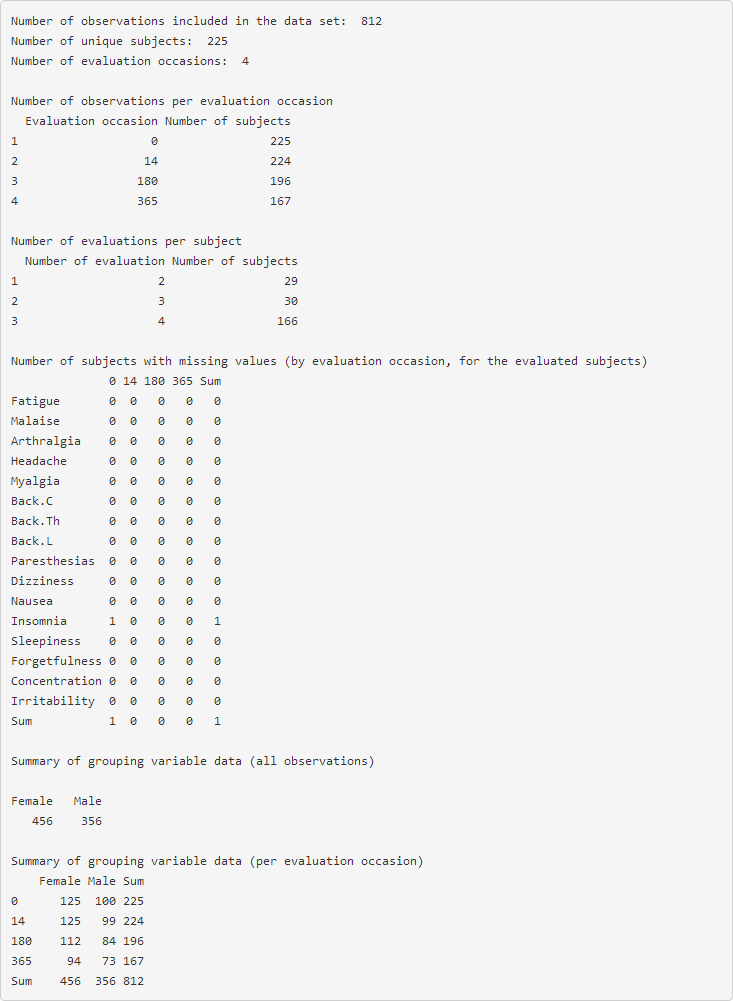

Supplement: S1 Fig — Output on the Data Summary tab, showing basic summary of the data. (TIF) [file pone.0121760.s004.tif]

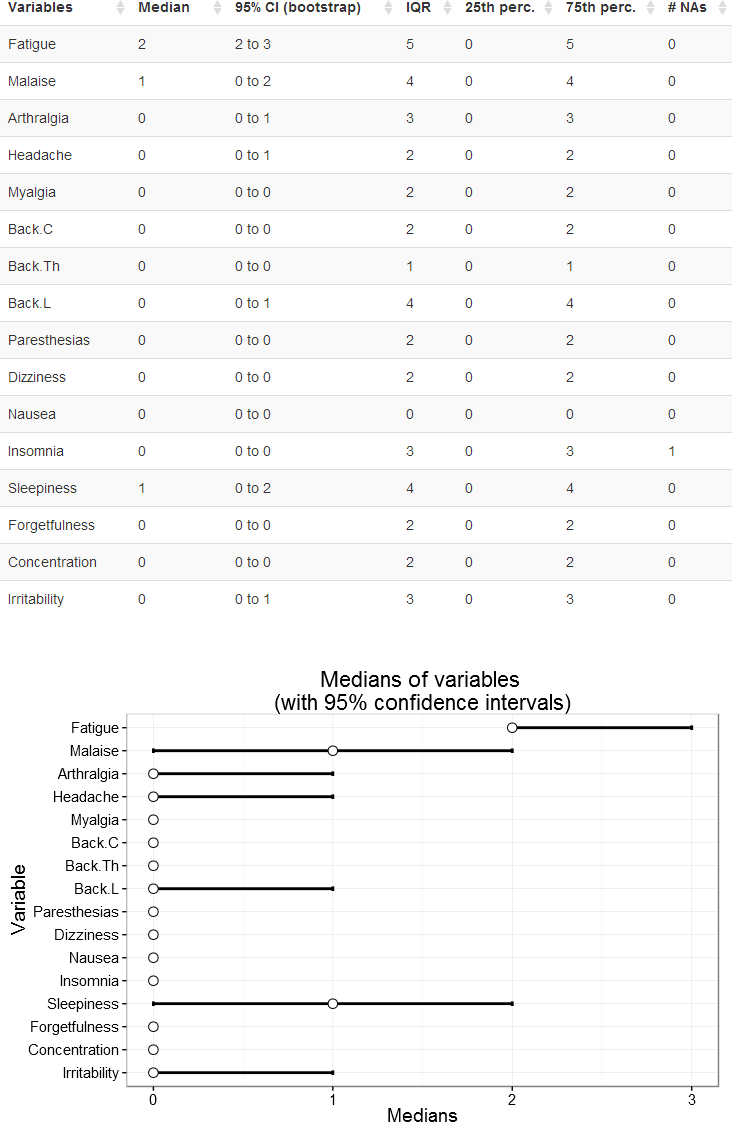

Supplement: S2 Fig — The table displays the descriptive statistics for the intensities of symptoms at baseline (medians, interquartile ranges, 25% and 75% percentile, number of missing values), and 95% confidence intervals for the medians based on bootstrap. The medians and 95% CI are also graphically displayed. (TIF) [file pone.0121760.s005.tif]

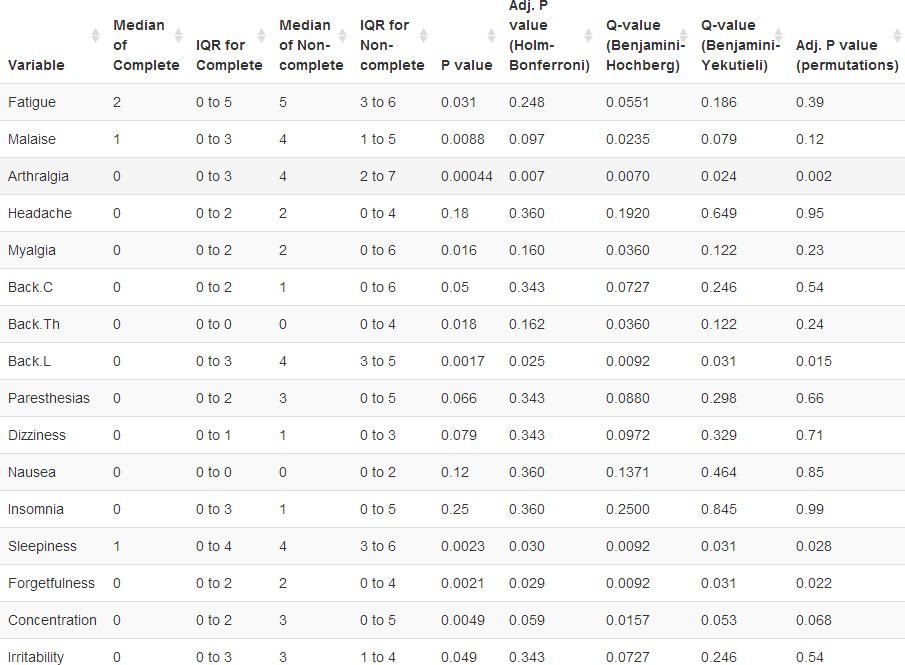

Supplement: S3 Fig — The table displays the summary statistics for the intensity of symptoms at baseline for groups defined by the response to treatment at last evaluation. The proportions are compared, unadjusted and adjusted P values and Q values are provided (see text for details). (TIF) [file pone.0121760.s006.tif]

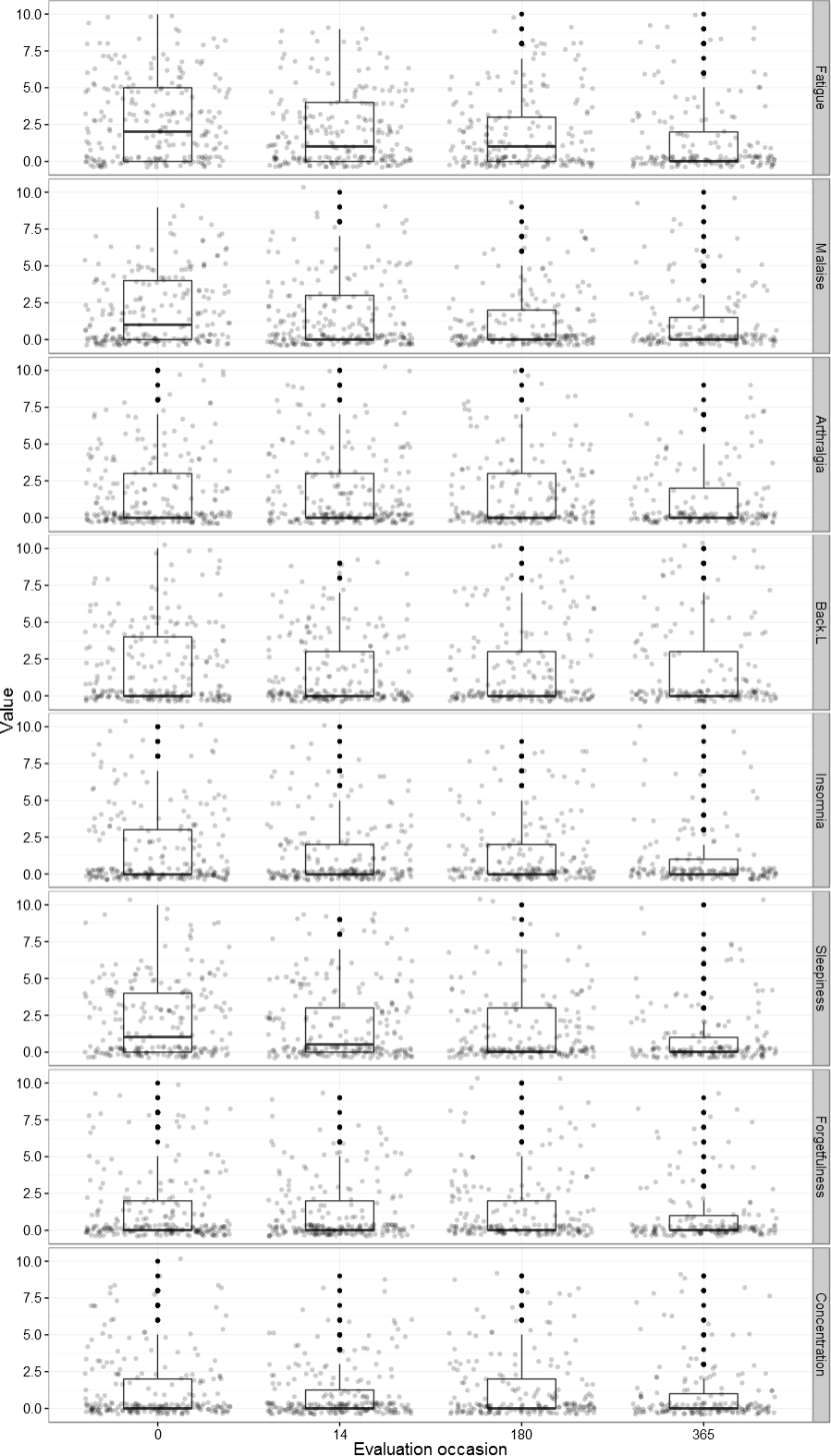

Supplement: S4 Fig — Boxplots displaying the intensities of symptoms reported at each evaluation occasion. (TIF) [file pone.0121760.s007.tif]

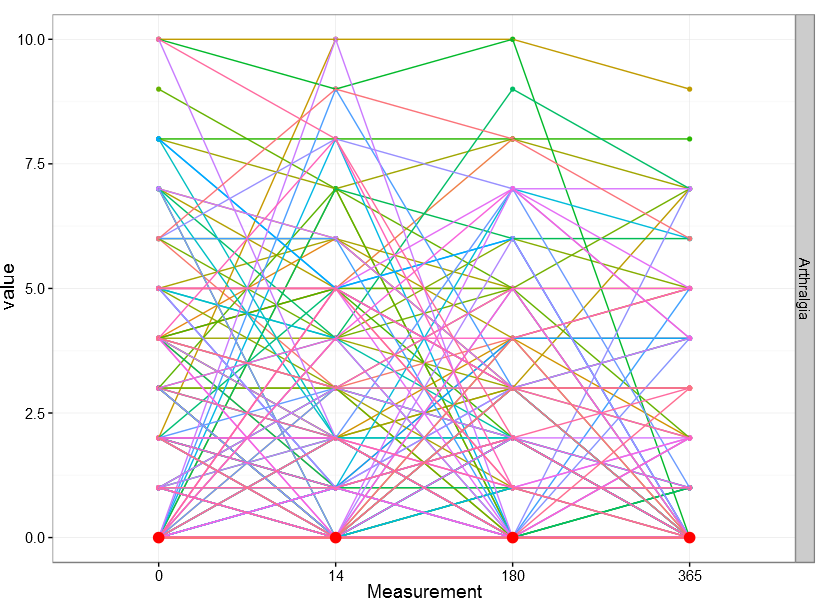

Supplement: S5 Fig — The intensity of arthralgia is shown for each patient and evaluation occasion (horizontal axis). Each line connects the values for the same patient. (TIF) [file pone.0121760.s008.tif]

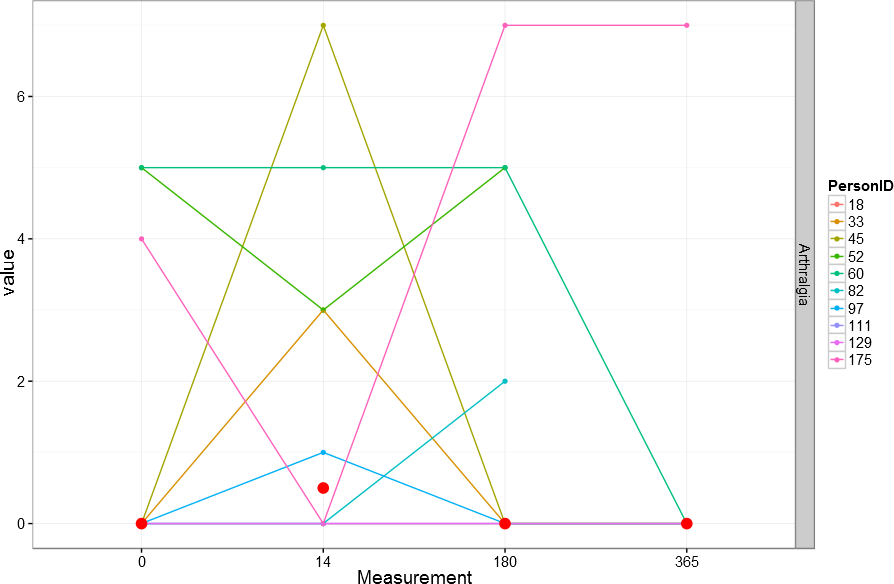

Supplement: S6 Fig — The intensity of arthralgia is shown for a subset of 10 patients. (TIF) [file pone.0121760.s009.tif]

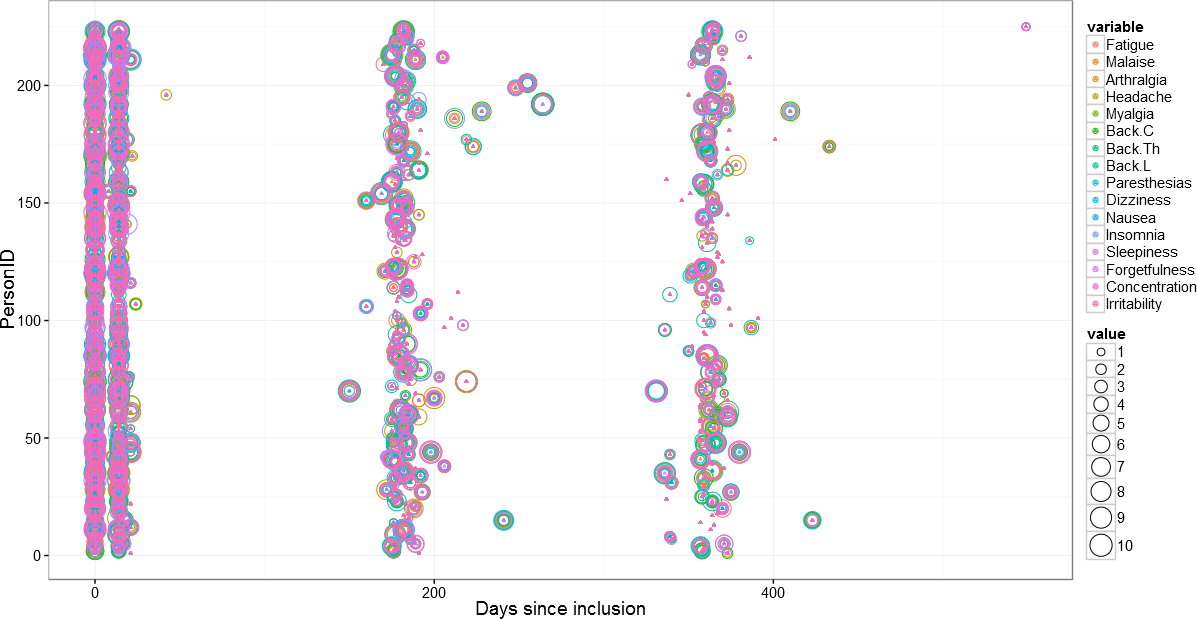

Supplement: S7 Fig — Intensities of reported symptoms are represented by different sizes of circles. Each line represents a patient. The horizontal axis represents days since inclusion in the study. The size of the bubbles is proportional to the intensity of the symptoms. (TIF) [file pone.0121760.s010.tif]

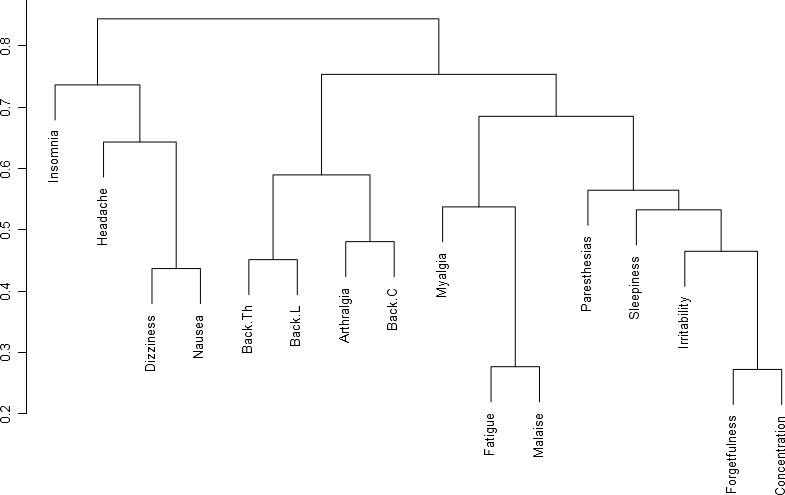

Supplement: S8 Fig — The intensities of the symptoms at baseline evaluation are grouped using hierarchical clustering. The results are displayed using a dendrogram. (TIF) [file pone.0121760.s011.tif]

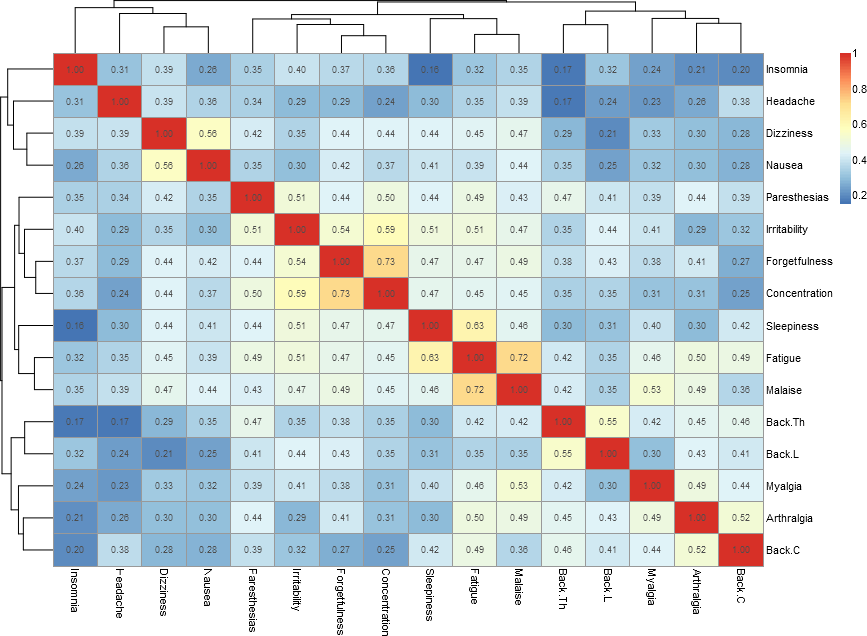

Supplement: S9 Fig — Graphical display of the Spearman’s correlation of the intensity of the symptoms at baseline evaluation. (TIF) [file pone.0121760.s012.tif]

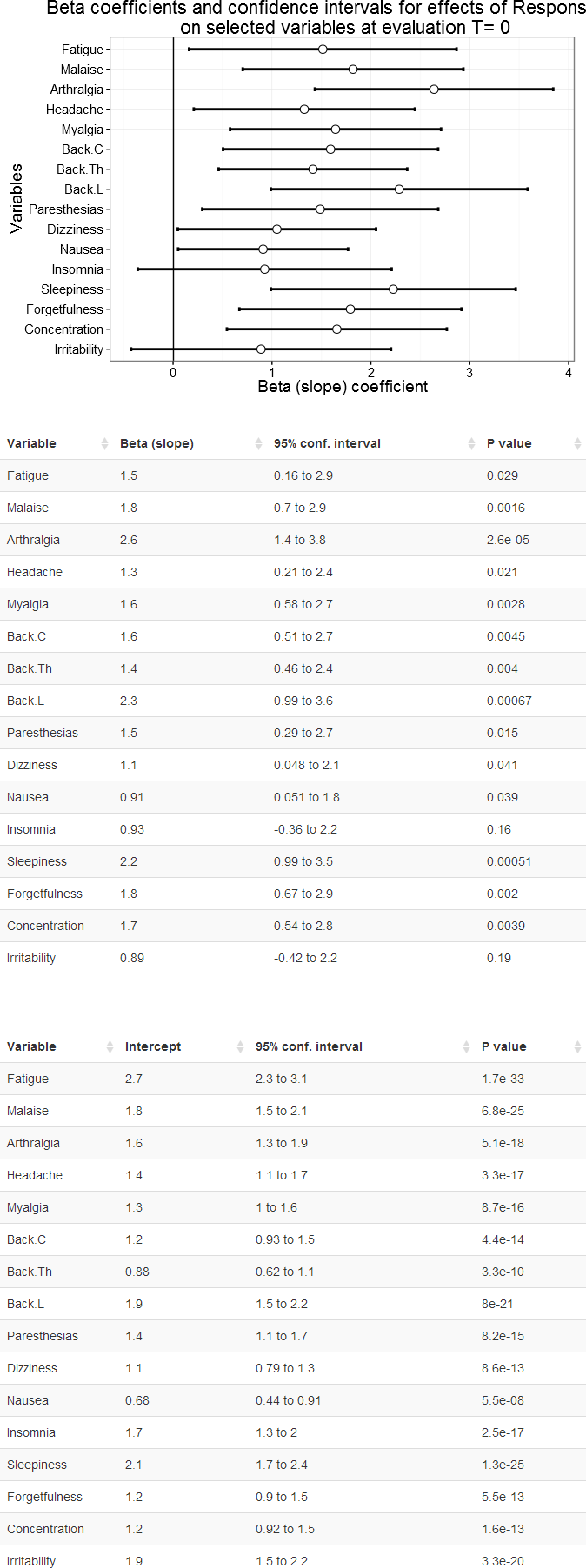

Supplement: S10 Fig — Estimates (with 95% CI and P values) obtained from the linear regression models where patient response at last available visit is used as a covariate and the intensity of the symptoms at baseline evaluation is the outcome variable. A separate model is fitted for each symptom; the estimated intercept of each model is also reported. The slopes and their 95% CI are also displayed graphically. (TIF) [file pone.0121760.s013.tif]

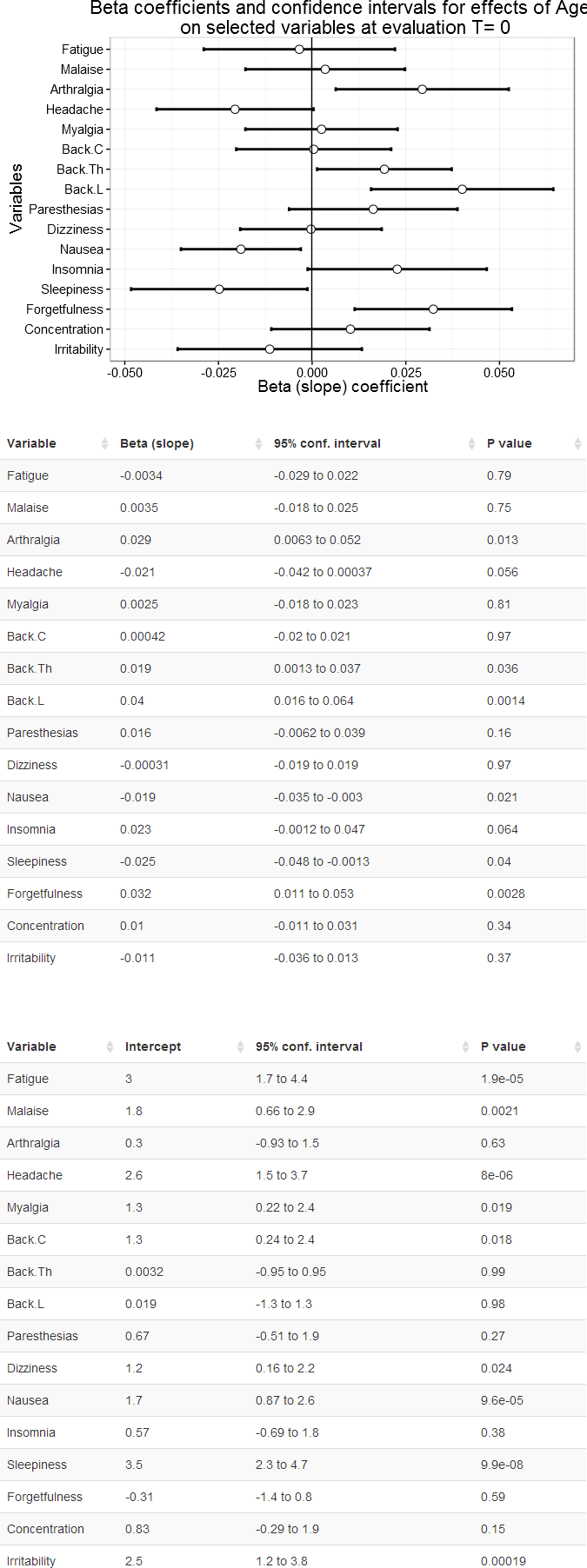

Supplement: S11 Fig — Estimates (with 95% CI and P values) obtained from the linear regression models where patient age is used as a covariate and the intensity of the symptoms at baseline evaluation is the outcome variable. A separate model is fitted for each symptom; the estimated intercept of each model is also reported. The slopes and their 95% CI are also displayed graphically. (TIF) [file pone.0121760.s014.tif]

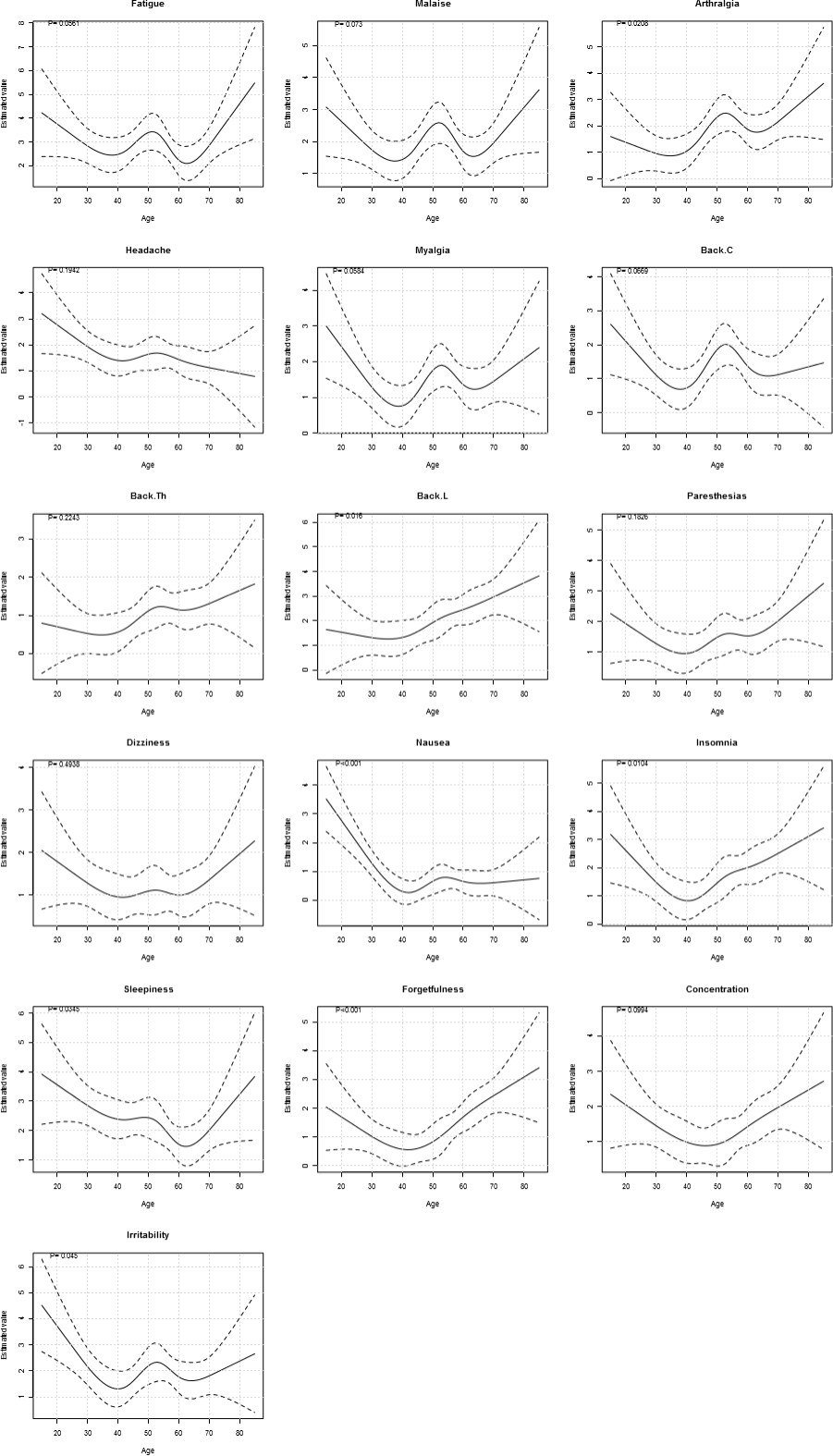

Supplement: S12 Fig — The graphs display the estimated associations between age (horizontal axes) and the intensities of symptoms at baseline evaluation occasion. Restricted cubic splines are used for modeling. (TIF) [file pone.0121760.s015.tif]

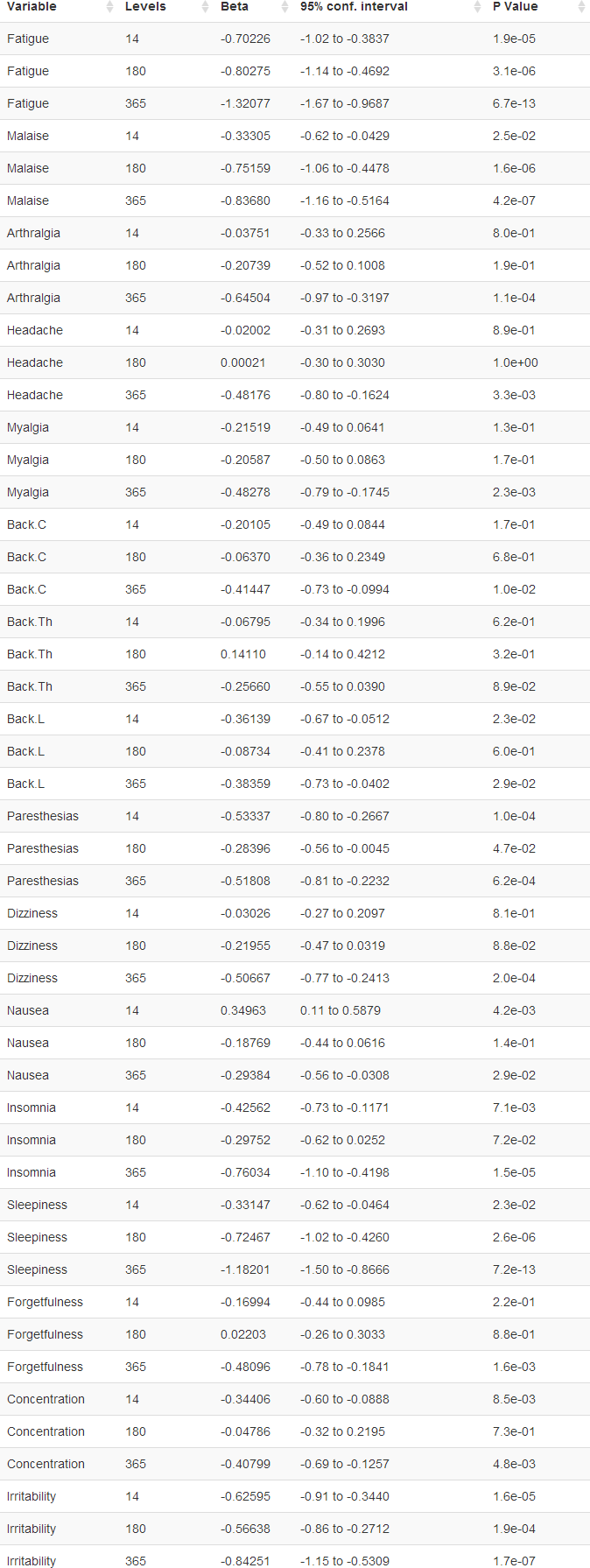

Supplement: S13 Fig — The table reports the estimated slopes obtained from linear regression models in which the association of the evaluation occasion (covariate) and intensity of symptoms (outcome) is evaluated. The multiple measurements from each patient are taken into account using a random intercept mixed model. (TIF) [file pone.0121760.s016.tif]

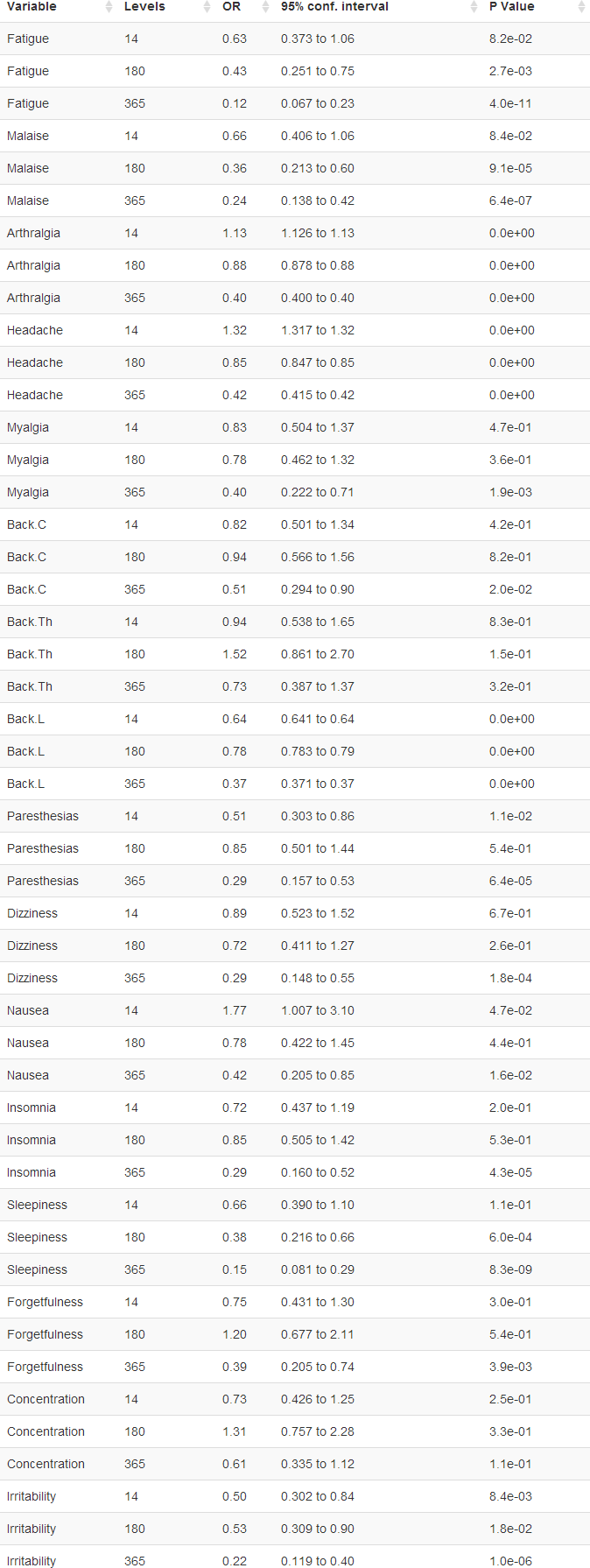

Supplement: S14 Fig — The table reports the estimated odds ratios obtained from logistic regression models in which the association of the evaluation occasion (covariate) and presence of symptoms (outcome) is evaluated. The multiple measurements from each patient are taken into account using a random intercept mixed model. (TIF) [file pone.0121760.s017.tif]

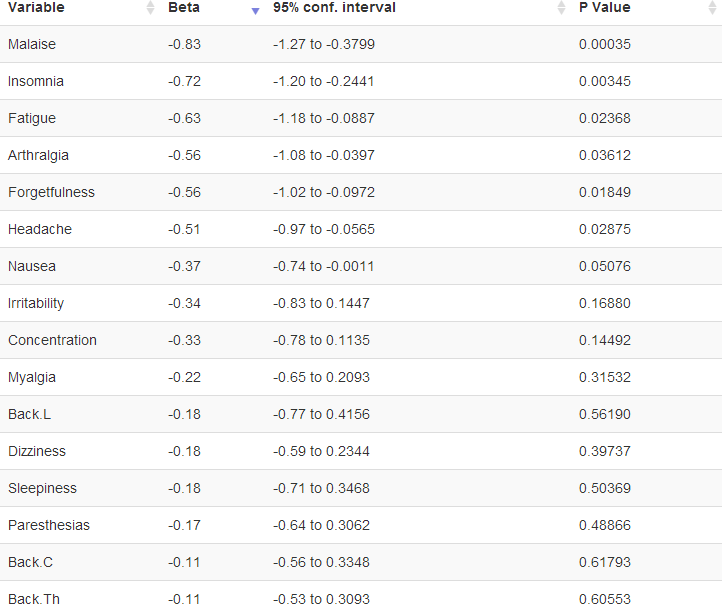

Supplement: S15 Fig — The table reports the estimated slopes for the effect of covariate (sex) on the outcome (intensities of symptoms). A second covariate (evaluation occasion) is included in the model. The multiple measurements from each patient are taken into account using a random intercept mixed model. (TIF) [file pone.0121760.s018.tif]

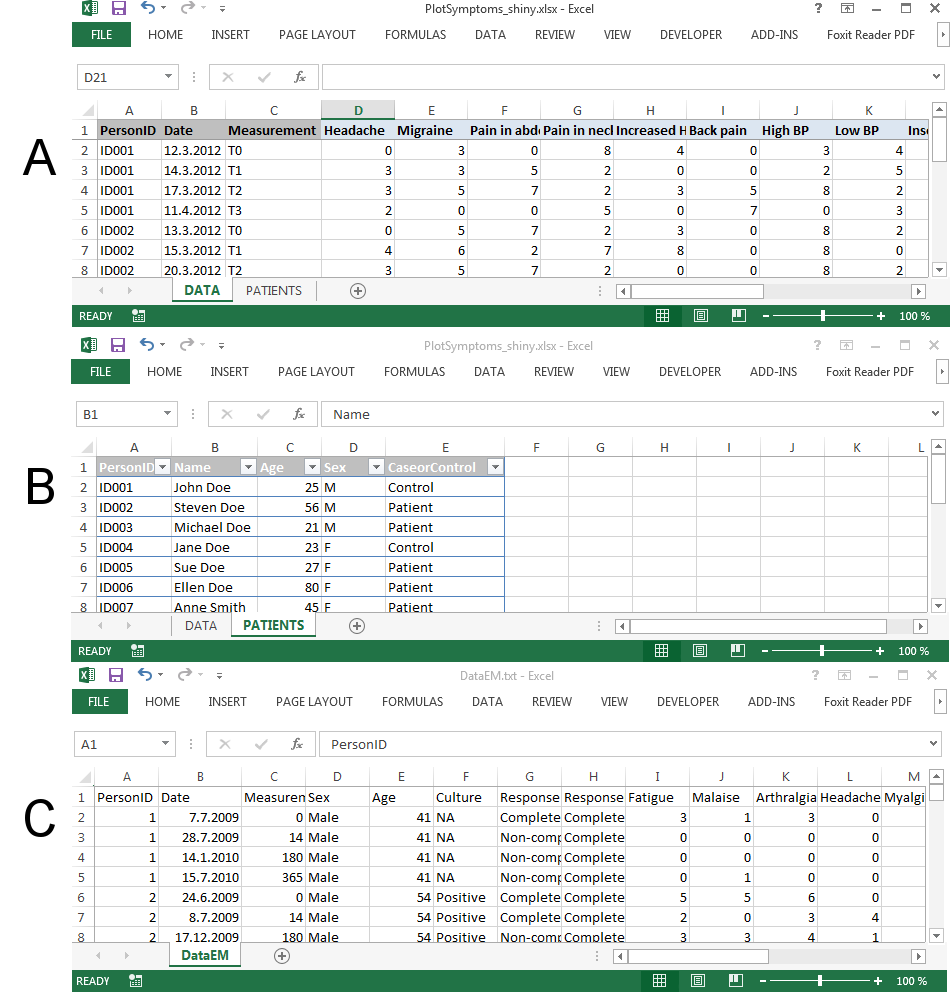

Supplement: S16 Fig — MS Excel sheets containing demo data in different formats. The panels are: A) Excel template file showing the DATA sheet, B) Excel template file showing the PATIENTS sheet, C) tab separated values file open in Excel. (TIF) [file pone.0121760.s019.tif]
